# The effect of bioelectric and manual impressions on quality of complete dentures and quality of life: A cross over pilot study

**DOI:** 10.1016/j.jobcr.2024.05.016

**Published:** 2024-05-29

**Authors:** Aditi Nanda, Modhupa Ghosh, Smiti Bhardwaj, Mahesh Verma, Harsimran Kaur

**Affiliations:** a Department of Prosthodontics, Centre for Dental Education & Research, All India Institute of Medical Sciences, New Delhi, India; b Department of Prosthodontics, Maulana Azad Institute of Dental Sciences, New Delhi, India; c Dental Implant Development Project, Maulana Azad Institute of Dental Sciences, New Delhi, India; d Guru Gobind Singh Indra Prastha University, Delhi, India; e Department of Dental Surgery, VMMC & Safdarjung Hospital, New Delhi, India

**Keywords:** TENS, Complete denture, Quality of life, Impression

## Abstract

**Purpose:**

To compare the quality of complete dentures and quality of life of participants rehabilitated by using TENS (Transcutaneous electric nerve stimulation) facilitated impression making with manual impressions.

**Material and methods:**

Ten completely edentulous participants were enrolled in the crossover, pilot study. Participants were randomized in 2 groups. Five participants in each group were rehabilitated by dentures fabricated with TENS facilitated definitive impression technique (group T) and conventional impression technique (group C). In group T, Bioelectric border molding was done for the participants, that uses electric stimulation of the nerves supplying the muscles. In group C, incremental border molding using modeling plastic impression compound was carried out. Participants in each group used the dentures for 3 months. After 3 months, OHIP-EDENT questionnaire responses were obtained from the participants to observe the oral health related quality of life. A dental specialist recorded denture quality by Kapur scoring criteria. After one month wash period, the treatment was swapped between the groups. OHIP-EDENT scores and Kapur score were recorded for the alternate dentures after 3 months of use. Descriptive analysis was followed by Mann Whitney test to compare the overall scores between group T and group C for OHIP-EDENT, the scores for individual domains of OHIP-EDENT, and Kapur score for denture evaluation (α = 0.05).

**Results:**

The overall OHIP-EDENT scores within each domain were less in group T when compared with the scores in group C and Kapur score for group T was more than group C. The difference was statistically significant i.e. *P* = 0.002 & 0.003 respectively.

**Conclusions:**

Less OHIP-EDENT scores in group T imply better perception of quality of life of individuals due to better performance of stomatognathic system. The higher Kapur scores in group T signifies better quality of dentures when TENS was used for definitive impression making.

## Graphical abstract

**Figure:**
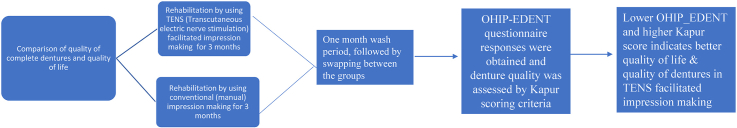

## 1 Introduction

Definitive impression making in complete denture fabrication is technically challenging procedure due to the variations imparted by the numerous documented techniques and variety of materials.^1^ The quality of definitive impression affects the outcome of complete denture rehabilitation considerably as it has a direct bearing on the quality of the prosthesis.^2^^,^^3^ Anatomically, the muscles surrounding the denture bearing area of maxilla and mandible have an important role in determining the border seal as well as in establishing the functional extent of the complete denture prosthesis. Border molding has conventionally been performed either by the operator, the patient, or both during this procedure. However, these methods carry an inherent degree of subjectivity due to the human element of performance. The variability makes it difficult to master a technique, especially by the operators who are in the early phase of learning.^4^, ^5^, ^6^, ^7^, ^8^

Along with the manual methods of impression making in completely edentulous scenarios, digital impressions by using intraoral scanners have been attempted to develop an analogue of the denture bearing area. However, lack of a definitive anatomic landmark in completely edentulous arches makes the digital impressions less accurate than conventional impressions. The accuracy is further reduced in situations with increased mobility of soft tissue.^9^, ^10^, ^11^, ^12^

Denture quality has commonly been related to denture retention and stability.^13^^,^^14^ Numerous operator based assessments have been suggested to evaluate denture quality with the understanding that denture quality has a direct impact on patient satisfaction and quality of life of the patients.^15^^,^^16^

Kapur scoring criteria is a method that provides a criterion for classifying the overall clinical quality of a complete denture.^17^ Dentures are clinically appraised, and scoring is done for retention and stability on a scale for each denture base individually. For each evaluation, minimum score that can be imparted for both retention and stability is 0, maximum score that can be imparted for retention is 3, and maximum score for stability is 2. The sum of scores provides the clinical rating and quality of dentures (good, satisfactory, or poor).^17^

Oral specific health status measures have been advocated by numerous authors to assess the outcome of interventions and oral health related quality of life changes after implementation of prosthetic rehabilitation procedures.^18^, ^19^, ^20^ OHIP is an instrument that was developed in 1994 by Slade and Spencer for the assessment of quality of life.^21^ The abridged version of OHIP for edentulous patients was developed in 2002, in the UK. This questionnaire has 19 questions that are distributed in 7 subscales: functional limitation, physical pain, psychological discomfort, physical disability, social disability, and handicap. Through these questions, this tool helps to measure the changes in the perception of oral health after prosthetic rehabilitation of a completely edentulous individual is completed.^22^, ^23^, ^24^, ^25^, ^26^

Variations in the concepts of manual and digital methods of impression making for completely edentulous individuals makes it impossible to achieve universal application of a single technique in all clinical situations. Consequently, there is a need to continuously improve on the alternative methods that may have greater applicability in challenging clinical scenarios. Bioelectric border molding is a procedure that uses electric stimulation of the nerves supplying the muscles.^27^ Electric nerve stimulation of muscle activity can be done by supplying electric current over the skin surface. Such a procedure is known as transcutaneous electric nerve stimulation (TENS).^28^ To the authors’ knowledge, the effectiveness of TENS in definitive impression making for complete denture fabrication has not been attempted in the past.

The purpose of this clinical study was to evaluate and compare the quality of dentures and quality of life of participants rehabilitated by using TENS during impression making and conventional impression during complete denture fabrication. The null hypothesis were that statistically no significant difference would be observed in the Kapur score and OHIP-EDENT of individuals rehabilitated by using TENS during impression making and conventional impression during complete denture fabrication after 3 months of use of dentures.

## 2 Material and methods

This prospective, cross over study was started after obtaining ethical clearance from the Institution Ethical Committee (Reference no: IEC18/MAIDS/2016). The informed consent was obtained for experimentation with human subjects before initiation of the study. Since previous clinical study results were not available during the establishment of the study design, biometric sample size calculation could not be done. A convenience sample size of 10 in each group was selected for this pilot study. Participants between the age of 45–80 years, with completely edentulous maxillary and mandibular arch, observing a minimum of 1 month duration of complete edentulism without prosthetic rehabilitation, and well versed with English language, were included in the study.

Participants belonging to class I and II of the Prosthodontic diagnostic index classification proposed by the American College of Prosthodontists were include.^29^ Participants with temporomandibular disorders, myopathies, motor neuron diseases, psychiatric or psychological disorders, a history of radiotherapy for the head and neck region, terminal systemic diseases immunocompromised, smokers, pregnant women, medically compromised patients, and with compromised oral hygiene were excluded. Before starting treatment, the methodology was explained in detail to each participant for both the treatment protocols, and written informed consent was received. All the procedures were carried out by a single operator to minimize variation in treatment rendered.

Since the design of the study was cross over, 5 participants were rehabilitated by dentures fabricated by using TENS (group T). The remaining 5 participants were rehabilitated by dentures fabricated with the conventional impression procedure (group C). The participants were randomly selected into intervention (group T) and control (group C) groups by simple, computer-generated randomization, with 1:1 allocation ratio. Implementation of randomization was done by a staff nurse, and allocation was done by using sequentially numbered opaque sealed envelopes before patient recruitment. Participants in each group used the dentures for 3 months.

After 3 months, response from the OHIP-EDENT questionnaire was obtained from the participants.^19^^,^^22^ Maxillary and mandibular complete dentures were clinically appraised by a dental specialist for stability and retention by using Kapur score. The minimum score that could be imparted for both retention and stability was 0, maximum score that could be imparted for retention was 3, and for stability was 2.^15^^,^^24^

A 1 month time period was then observed as wash period, during which the participants were asked not to use any prosthesis. After the wash period, the treatment was swapped between the groups. The participants in each group were asked to provide OHIP-EDENT after 3 months of using the alternate denture. Denture quality was also assessed after 3 months by using Kapur score as described before.

A custom tray was fabricated for each arch of each participant on the diagnostic cast, after adaptation of a spacer of 1.5 mm thickness. For impression of each arch, similar steps were followed within each group.

**Fig. 1:**
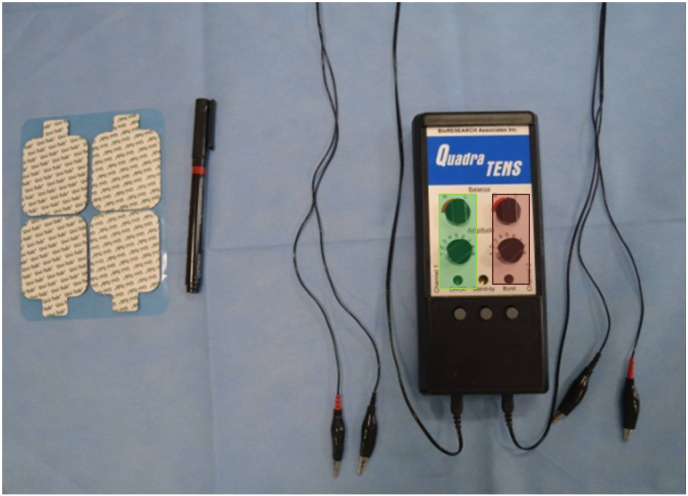
TENS machine (Quadra-TENS, Bio Research Associates)

**Fig. 2:**
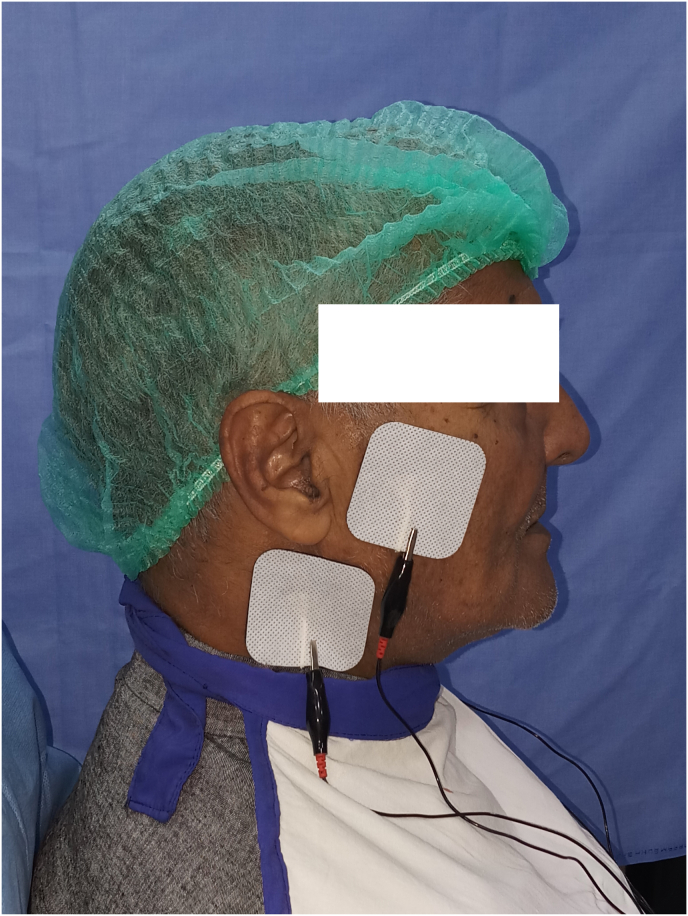
Electrodes connected to pre-auricular region and posterior triangle of neck

Low frequency TENS (10 Hz) with 4 channels as previously described was used to make definitive impressions in group T.^27^ TENS machine (Quadra-TENS, Bio Research Associates) [Fig. 1] was operated in continuous mode with an intensity of 20 mA. The channels were connected to the electrodes. The electrodes were clipped to the adhesive pads that were adhered to the skin as shown in Video 1. This enabled transcutaneous electric stimulation of nerves. Out of the 4 channels, 2 channels were used to stimulate the nerves in preauricular region for left and right sides (Channel 1), and 2 channels were used to stimulate nerves in posterior triangle of neck for left and right sides (Channel 2). Electrodes connected to channel 1 stimulated the facial nerve and mandibular division of trigeminal nerve. Electrodes connected to channel 2 stimulated the accessory nerve and increased the parasympathetic activity by stimulation of the vagus nerve [Fig. 2]. For both maxillary and mandibular impression, the muscles were activated by using TENS in group T. Tray adhesive was applied to the borders of the tray (3 M Dental Products, St. Pau, MN) and border molding of the custom tray for definitive impression was accomplished by applying heavy viscosity polyvinyl siloxane (Reprosil VPS Heavy body, Dentsply Sirona) around the periphery of the impression tray. The electrodes were connected to the TENS machine and the tray was seated in the mouth. Once the impression material had polymerized, the tray was retrieved, spacer wax was removed, tray adhesive was reapplied, and low viscosity polyvinyl siloxane impression material (Reprosil VPS Light body, Dentsply Sirona) was used to make the definitive impression as shown in Video 2. During border molding and impression making, the trays were held in position by fingers of the operator in the mouth. No manual or functional molding was done for maxillary definitive impression. Patient was asked to swallow during mandibular impression making.

For the conventional impression technique, border molding of the custom trays was accomplished by incremental technique by using modeling plastic impression compound (Pinnacle Tracing Sticks, DPI). Each individual segment was molded by combination of manual molding by the operator and functional movements performed by the patient. After completion of border molding, spacer wax was removed, tray adhesive was applied to the impression tray and the molded borders, and definitive impression was made by using low viscosity polyvinyl siloxane impression material (Reprosil VPS Light body, Dentsply Sirona).

After obtaining the final impression, in both the groups, jaw relation was recorded and teeth arrangement was done on the semi-adjustable articulator to establish balanced occlusion. Try-in was done to the satisfaction of both clinician and participant. Dentures were fabricated following the conventional procedure. After 3 months of use of dentures, OHIP-EDENT and Kapur score was obtained for each participant.

The data were collected for OHIP-EDENT and Kapur score, and analyzed with a statistical software program (Stata 16.0; StataCorp LLC). Quantitative variables were summarized as mean and standard deviation for each of the 2 groups. Approximate normality was tested by using the Shapiro-Wilk test. Since the data were not normally distributed, Mann Whitney test was used to compare the overall scores obtained for OHIP-EDENT, the scores for individual domains between group T and group C, and Kapur score for denture evaluation (α = 0.05).

## 3 Results

Descriptive data for OHIP-EDENT are seen in Table 1. The overall OHIP-EDENT score values were less in group T when compared with the value of scores in group C, and the difference was statistically significant (*P* = 0.002) as seen in Table 1. For each domain as well, the OHIP-EDENT score was less in group T when compared with group C. A statistically significant difference was observed on comparing the OHIP-EDENT scores between the 2 groups for each domain (*P* = 0.016 for functional limitation, *P* = 0.010 for physical pain, *P* = 0.038 for psychological discomfort, *P* = 0.038 for physical disability, *P* = 0.013 psychological disability, *P* = 0.007 for social disability, and *P* = 0.001 for handicap).

**Table 1:** Descriptive data and comparison between group T and group C for overall OHIP-EDENT score and OHIP-EDENT scores for each domain.

	OHIP-EDENT	FL	PP	PD	Ph D	Ps D	SD	H
Group T	8.80 ± 3.15	1.5 ± 0.52	1.3 ± 0.82	1.3 ± 0.48	1.2 ± 0.78	0.9 ± 0.56	1.4 ± 0.69	1.2 ± 0.42
Group C	15.20 ± 1. 99	2.3 ± 0.48	2.3 ± 0.4	2.0 ± 0.66	2.1 ± 0.73	1.8 ± 0.63	2.4 ± 0.52	2.3 ± 0.48
*P* value	*P* = 0.002	*P* = 0.016	*P* = 0.010	*P* = 0.038	*P* = 0.038	*P* = 0.013	*P* = 0.007	*P* = 0.001

FL, Functional limitation; PP, Physical pain; PD, Psychological discomfort; Ph D, Physical disability; Ps D, Psychological disability; SD, Social disability; H, Handicap.

Descriptive data for Kapur score are seen in Table 2. The overall Kapur score for group T was more when compared with the value of scores in group C and the difference was statistically significant (*P* = 0.003) as shown in Table 2. The scores for retention were more in group T when compared with group C on individual evaluation of the dentures, although the difference was statistically not significant (*P* = 0.362 for maxillary denture, *P* = 0.064 for mandibular denture). The scores for stability were more in group T when compared with group C when dentures were individually evaluated, and the difference was statistically significant (*P* = 0.032 for maxillary denture, *P* = 0.032 for mandibular denture).

**Table 2:** Descriptive data and comparison between group T and group C for Kapur score.

Descriptive data of Kapur scores	KSR maxillary	KSS maxillary	KSR mandibular	KSS mandibular	Overall KS
Group T	2.8 ± 0.63	3.1 ± 0.88	2.9 ± 0.32	2.8 ± 0.92	11.6 ± 2.07
Group C	2.5 ± 0.52	2.1 ± 0.83	2.4 ± 0.52	1.8 ± 0.52	8.8 ± 1.07
Comparison between groups (*P* value)	*P* = 0.362	*P* = 0.032	*P* = 0.064	*P* = 0.032	*P* = 0.003

KS, Kapur Score; KSR, Kapur Score for Retention; KSS, Kapur Score for stability.

## 4 Discussion

The results of the study led to the rejection of both the null hypotheses. The difference in the OHIP-EDENT and Kapur score of individuals rehabilitated by using TENS during definitive impression making and conventional impression during complete denture fabrication were observed to be statistically significant after 3 months of use of dentures.

The overall OHIP-EDENT score on using complete dentures fabricated by using TENS was less when compared with conventional method of impression making. OHIP measures the self-reported dysfunction, discomfort, and disabilities imputed to the stomatognathic condition.^18^, ^19^, ^20^, ^21^, ^22^, ^23^ Lesser scores of OHIP-EDENT imply better perception of quality of life of individuals due to greater satisfaction of functions of stomatognathic system. Additionally, the lower OHIP-EDENT scores for group T in all the 7 domains when compared with group C imply a positive subjective feedback suggestive of contentment with quality of life due to better oral health status.

The overall Kapur score with complete dentures fabricated by using TENS was more when compared with the score observed with complete dentures fabricated by conventional method of impression making. A higher Kapur score implies better quality of dentures than the quality observed with lower score.^15^^,^^26^ The statistically significant difference in stability of dentures between group T and group C implies greater impact of muscle drape as compared to other factors involved in influencing stability of the prosthesis. Higher accomplishment of border seal and hence retention within group T can also be contributory to enhanced stability.^14^

Thus both the outcome parameters, including the quality of life as perceived by the participants and quality of prosthesis as appraised by professionals, were enhanced in cases rehabilitated by using TENS in definitive impression during complete denture fabrication. The possible reason for this observation can be attributed to purely physiological muscle molding due to nerve stimulation through the electric stimulus.

The electrodes in the preauricular region brought about stimulation of the facial nerve and the mandibular division of trigeminal. The muscles that are recorded due to stimulation of the facial nerve include the buccinators, orbicularis oris, and levator labii superioris. Stimulation of mandibular division of trigeminal nerve brings about activation of tensor veli palitinus. In the posterior triangle of neck, the stimulation of accessory nerve elicits the action of palatoglossus, palatopharyngeous, and levator veli palatine. Additionally, the increase in the parasympathetic activity due to the stimulation of the vagus nerve leads to a generalized calming effect on the individual.

Omission of manual manipulation of tissues circumvents the risk of over shortening or over extension of the borders of the impression, and thus physiologically limits the denture border. The lack of direct manual intervention also overcomes the dependency on patient compliance or operator performance to mold the muscles of the denture bearing area.

The study also reveals the merits of using TENS in definitive impression making during the fabrication of complete dentures. Improved oral health related quality of life as reflected in overall scores and scores of all domains suggest an improvement in everyday lives of individuals due to an eventual improvement in the performance of oral function, social activity, and emotional life of the individual. Improved quality of dentures can promote longevity of the prosthesis as well as tissue health of the denture bearing area. A favorable outcome in group T as reflected in the comprehensive evaluation of patient and professional feedback implies that the use of TENS in making definitive impressions is a promising alternate that can be beneficial for improving everyday lives of edentulous individuals and the overall success of the rehabilitation.

Despite the favorable outcome that has been observed by using TENS for impression making in complete denture fabrication, TENS should not be considered a definitive method of impression making. This is because, the study has been conducted on a small participant group and will need to be supported by a larger sample size. A longer follow up to assess maintenance of the effect of dentures on oral health related quality of life and quality of dentures beyond 3 months is also suggested. Additionally, the study has been conducted only on patients belonging to class I and class II of Prosthodontic diagnostic index, and the results cannot be generalized to all the class of patients. Lastly, patient feedback is subject to variable experience, as some patients can adapt better to dentures than other patients.^1^^,^^2^

The main advantage of impression making utilizing TENS is that it assists in physiological muscle trimming without any over-shortening or overextension of the borders. In addition, the manual manipulation of tissue either by operator of by the patient is highly reduced-which is chiefly responsible for variability and unpredictability of the impression in other techniques. It is a single step procedure. It presents with Consistency of results, improved outcome and patient satisfaction. The pressureless/mucostatic technique makes it an extremely useful technique in excessively resorbed cases and in cases with inflamed/injured tissues. This technique provides a relaxed ambiance to the patient during impression making. There are few limitations of the study, use of TENS requires special equipment to be procured and is not recommended on broken skin areas or wounds, tumor sites, in patients with pacemakers, and in pregnant women. A limitation noticed while using TENS is the lack of the impact of TENS on hypoglossal nerve. Lastly, muscle physiology is not the sole factor that can affect the outcome of patients' perception and clinicians’ appraisal. Other confounding factors that impart success to prosthesis cannot be overlooked.

A possible application of use of TENS can however be in clinical situations of compromised ridge morphological conditions where complete dentures may be the recommended treatment option and in cases where patients have limited comprehension of following operator instructions (such as deaf patients). This is because the physiological capture of muscles of denture bearing area can possibly eliminate the unpredictability of clinical outcome.

## 5 Conclusions

Based on the findings of this pilot clinical study, the following conclusions were drawn:

1. When compared to conventional impression making procedure, the use of TENS during definite impression making in complete denture fabrication imparted enhanced patient feedback, reflecting greater impact on oral health related quality of life and a better clinical appraisal, reflecting higher denture quality.
2. TENS can be used as a bioelectric tool to facilitate impression making during complete denture impression in select clinical situations.

## Declaration of competing interest

None.

## Funding/support statement

Authors declare that there is no funding or support for the research, work, writing and editorial assistance from internal or external agencies, including commercial companies.

## Appendix A Supplementary data

Multimedia component 1 Attachment of electrodes Multimedia component 1

Multimedia component 2 Definitive impression with TENS Multimedia component 2
